# Plain X-ray, computed tomography and magnetic resonance imaging findings of telangiectatic osteosarcoma: a case report

**DOI:** 10.4076/1757-1626-2-7833

**Published:** 2009-09-16

**Authors:** Vasilios Skiadas, Vasilios Koutoulidis, Andreas Koureas, Lia Moulopoulos, Athanasios Gouliamos

**Affiliations:** 1 Department of Radiology, 401 General Army HospitalMakedonias 41, Ilion, 13123, AthensGreece; 2 Department of Radiology, University of Athens, Aretaieion HospitalVas. Sofias 76, AthensGreece

## Abstract

An 18-year-old male patient presented with chronic nonspecific pain of three months located at his left proximal tibia. The patient was admitted to our department for plain X-ray, computed tomography and magnetic resonance imaging examination. Plain X-ray and computed tomography revealed a geographic lytic lesion at the medial aspect of the proximal tibia. Biopsy of the lesion showed telangiectatic osteosarcoma. Image findings of all modalities are presented.

## Introduction

Telangiectatic osteosarcoma is an uncommon histopathologic subtype that represents 2.5 -12% of all osteosarcomas [1]. It is difficult to be diagnosed as it is almost entire lytic and often much less aggressive radiographically than conventional osteosarcoma. [2] Radiologic findings of such a case in plain X-ray, CT and MRI are presented.

## Case presentation

An 18-year-old male patient from Greece was admitted to our department due to chronic nonspecific knee pain (for the last 3 months) for a plain X-ray and an MRI examination.

Clinical examination two weeks prior to MRI revealed pain at the medial compartment and a meniscal tear was considered the most probable diagnosis, as the patient was also a semi-professional footballer. Physical examination revealed no skin stigmata, orbital pathology or other pathologic conditions. There was no history of previous trauma and his medical history was unremarkable. Laboratory findings were also unremarkable. No medication at the time of the examination except occasionally anti-inflammatory agents. The patients did not smoke or drink. From family history, and according to information from patient’s father, his mother died 3 years ago (at the age of 42) from an ovarian cancer and his little brother at the age of 3 from a brain tumour (no more data were available).

**Figure 1a and b.**
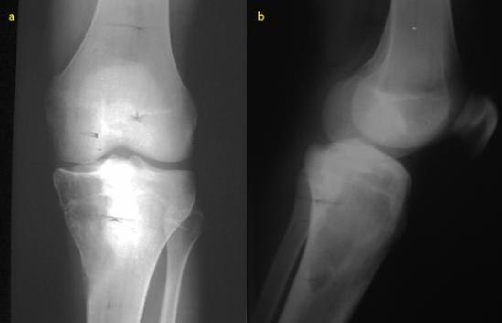
Plain X-ray examination of LT knee (anteroposterior and lateral view).

**Figure 2a and b.**
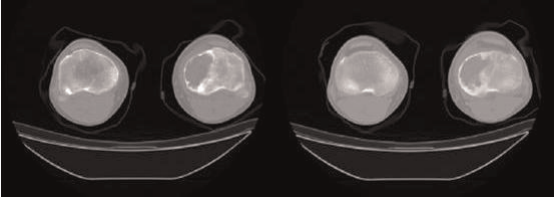
CT examination (sequential images at the level of the lesion) reveals slight bone expansion, cortical thinning and endosteal scalloping, indistinct margins and calcifications inside the lesion.

**Figure 3.**
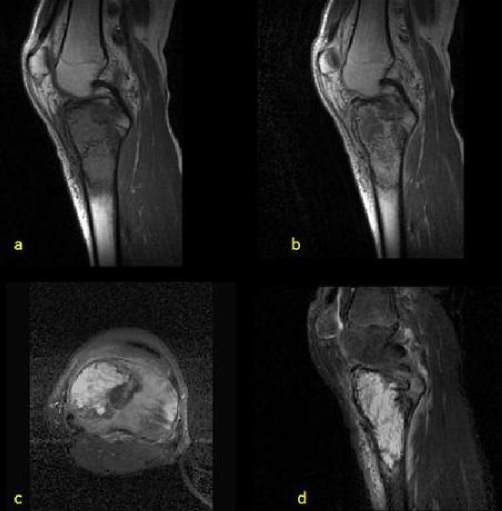
Sagittal T1-weighted image **(a)**, sagittal contrast enhanced T1-weighted image **(b)**, axial **(c)** and sagittal **(d)** T2-weighted images reveals a predominantly cystic lesion at the medial aspect of the proximal tibia with septations, fluid-fluid levels and a thick enhancing periphery. Bone marrow edema at the lateral aspect of the tibia and at the subcutaneous tissues also noted.

Plain X-ray (Figure 1a,b) revealed a geographic lytic lesion at the medial aspect of the proximal tibia with no evidence of soft tissue mass or internal calcifications. Small periosteal reaction demonstrated at the lower aspect of the lesion. CT examination (Figure 2) confirmed the findings of plain X-ray and showed a lytic geographic lesion with wide zone of transition at its lateral aspect causing slight expansion and thinning of the cortical bone, scalloping of the endosteum, no soft tissue extraosseous mass. Internal amorphous calcifications were demonstrated inside the lesion. MRI contrast enhanced examination (Figure 3a-c) revealed a lesion of fairly homogenous low signal intensity at T1-weighted sequences, mainly cystic lesion with fluid-fluid levels at T2-weighted images. After contrast agent administration (Figure 3d) enhancing viable tissue was detected mainly at the periphery of the lesion. CT of the lungs, MRI examination of the rest of the tibia and bone scintigraphy was negative. Biopsy of the lesion showed telangiectatic osteosarcoma. The patient underwent initially chemotherapy followed by resection of the proximal tibia with prosthesis implantation. As far as the authors known, the patient had not been investigated for possible germline mutations of the tumour suppressor gene TP53.

## Discussion

Telangiectatic osteosarcoma is an uncommon histopathologic subtype that represents 2.5-12% of all osteosarcomas [1]. It was first described by Paget in 1854 as a medullary cancer of bone with extensive development of vessels and blood-filled cysts. Gaylord in 1903 used the term malignant bone aneurysm to describe a hemorrhagic poorly ossified telangiectatic osteosarcoma and in 1922, Ewing was the first to classify telangiectatic osteosarcoma as a distinct histologic variant, characterized by a malignant osteoid-forming sarcoma of bone with large blood-filled vascular channels [3].

Patient demographics, clinical symptoms and lesion locations are similar to those of conventional osteosarcoma. Previous studies showed a male predilection (M/F 2.1:1.0) and non-specific clinical symptoms of pain [1,4] It involves the femur (50%) or tibia (25%) most commonly [1]. It disseminates almost exclusively through the blood as bone lacks a lymphatic system, and the lung is the most common site followed by bone. Rare sites of involvement such as orbital metastasis have also been described [5].

In conventional X-ray telangiectatic osteosarcoma presents as a lytic metaphyseal lesion with geographic pattern of bone destruction and wide zone of transition [1]. Mild expansile remodeling of bone is common and likely accounts for the high incidence of pathologic fracture at presentation. Periosteal reaction, cortical destruction and associated soft-tissue mass were also frequent findings on radiographs. The presence of oblique parallel striations in the shaft is considered also a very special radiologic sign [4].

Bone scintigraphy often demonstrates peripheral increased radionuclide uptake with central photopenia (donut sign) due to the cystic portions of the lesion. CT confirms the plain X-ray findings and is the best modality for the depiction of internal calcifications. Low attenuation central portion of the lesion (lower than that of muscle) due to fluid-filled cystic spaces may also been seen. MRI demonstrates predominantly high signal intensity on T2-weighted in all cases. MR imaging evidence of hemorrhage either as foci of high signal intensity with all MR pulse sequences or as fluid levels was also seen in most cases (up to 90%) [1,3].

Pathologically, telangiectatic osteosarcoma consists of large hemorrhagic or necrotic cavities that compose most (-90%) of the tumor volume with viable high-grade sarcomatous cells around the periphery and septations of these spaces [3-14]. These histologic features are the cause of telangiectatic osteosarcoma often having been described as showing non-specific bone lysis on radiographs and simulating aneurysmal bone cyst. The soft tissue counterpart of telangiectatic osteosarcoma is uncommon in young patients and usually affects adults older than 50 years [6]. It presents similar radiographic appearance as intraosseous lesions.

## Conclusion

The knowledge of radiologic findings of telangiectatic osteosarcoma is important as its often much less aggressive appearance may lead to underdiagnosis of this lesion and delay in therapeutic management of the patient.
